# Multi-professional ethical competence in healthcare – an ethical practice model

**DOI:** 10.1177/09697330211062986

**Published:** 2022-02-25

**Authors:** Camilla Koskinen, Kari Kaldestad, Bente Dorrit Rossavik, Anne Ree Jensen, Grethe Bjerga

**Affiliations:** Faculty of Health Sciences, Department of Caring and Ethics, 56627 University of Stavanger, Stavanger, Norway; Department of Surgery, 60496 Stavanger University Hospital, Stavanger, Norway; Faculty of Health Sciences, Department of Caring and Ethics, 56627 University of Stavanger, Stavanger, Norway

**Keywords:** Ethics, competence, multi-professional healthcare, caring science, hermeneutic application research

## Abstract

**Introduction:**

The starting point is that ethical competence is the basis for ethical healthcare practices and quality of care. Simultaneously, there is a need for research and development from a holistic multi-professional perspective.

**Aim:**

The aim is to create a proposed model for multi-professional ethical competence grounded in clarified meanings and dimensions of ethical competence studied from a multi-professional healthcare perspective. The research questions are, what is ethical competence from a multi-professional healthcare perspective and what strengthens a multi-professional ethical healthcare practice?

**Research design:**

The research has a qualitative approach and hermeneutic application research design. Two groups with six participants from clinical practice and two scientific researchers in each group met four times for dialogue. Thematic analysis was used as an analysis method.

**Ethical considerations:**

The research is approved by the Declaration of Helsinki, the General Data Protection Regulations, and ethical permission was asked from the Norwegian Centre for Research Data (NSD).

**Results:**

The proposed model for multi-professional ethical competence encompasses a three-dimensional ethical value base that is underpinned by: Ethical attitude – a personal desire to do good; Ethical basis – the best for the patient as a common goal and Ethical culture – common goals and values in the organization. Multi-professional ethical competence is strengthened by: Reflection – to see with new wondering eyes; Time for talk – interdisciplinary teamwork and Leadership – an ethical role model and support.

**Discussion:**

Ethical competence has a strong link to the core of caring ethics and a deeper personal value base and attitude. Ethical competence involves the whole culture and is seen as a shared value base and a responsibility to do the best for the patient as a multi-professional team and organization. Ethical competence becomes active in healthcare practice by opening up for meaningful multi-professional talks and reflections.

## Introduction

Different ethical codes and guidelines have been developed to achieve ethical consciousness and to guide healthcare professionals’ actions. Courses in ethics, ethical committees, ethical rounds and ethical educations have been implemented into healthcare organizations to support healthcare professionals in handling ethically demanding situations and resolving ethical issues encountered in their daily practice.^1^ Healthcare professionals’ competence for ethical reflection on a systematic and regular basis is considered to improve healthcare patients’ experiences of integrity and dignity.^2^ According to Nordström and Wangmo,^3^ ethical competence is a crucial factor enabling healthcare professionals to make complex, value‐based decisions and to implement ethically sustainable care, and avoid ethically obscure actions in multi-professional healthcare practice. Milliken and Grace^4^ highlight the need for more research on developing ethical awareness and sensitivity among health professionals and on how health professionals develop ethical competence. The most common description of competence focus on that includes knowledge, skills, and attitudes.^5^ As this research has ethical competence, ethical awareness, and sensitivity in focus, competence includes a deeper view or the inner dimension that, according to Kulju,^6^ includes strength of character, ethical awareness, moral judgment, and willingness to do good.

According to Koskenvuori et al.,^7^ ethical competence studies focus mostly on nurses although the codes of ethics concern multi-professional groups. They highlight that it would be beneficial to study ethical competence from a multi-professional perspective as care predominantly takes place in a multi-professional context. Thus, there is a need for further research on ethical practice from a broad holistic and multi-professional perspective, that reflects the cooperative work of the different health professional groups and professionals’ experiences of ethical questions and ethical practice. Research from a holistic perspective on healthcare professionals’ ethical competence could provide new insights and understanding of ethical competence as a multidimensional phenomenon that promotes quality in healthcare practice. Simultaneously, there is a need for research and the development of ethical competence from a holistic multi-professional perspective. The aim of this research is thereby to create a proposed model grounded in clarified meanings and dimensions of ethical competence for further development of multi-professional ethical competence in healthcare.

## Background

A review of previous research shows that models developed for nurse’s ethical decision-making exist in different contexts. Park^8^ has reviewed 20 currently available structured ethical decision-making models and developed an integrated model on nurses’ competence in ethical decision-making in practice. Ågren Bolmsjö et al.^9^ have developed a teleological model for the analysis of everyday ethical situations to clarify perennial ethical problems in nursing home care for persons with dementia. Using the model in a specific context of care is a tool for focusing on the relevant aspects of a situation, not missing out on those that should influence the decisions, and gives a basis for a structured and joint reflection on ethical problems and documentation. Schlairet et al.^10^ describe a model for providing hospital-based ethics support services. The model provides a broad spectrum of care for the management of conflict and ethically difficult situations in healthcare and describes how an ethics consultation process is transformed according to the needs of ethically vulnerable patients. Kangasniemi et al.^11^ have developed a tentative model to understand patients’ roles and duties in nursing and healthcare. The ethical basis, prerequisites, outcomes and risks of patients’ duties are named in the model. The focus on models is often on the values and characters of nurses, on attitudes of care, decision-making in specific caring situations and outcomes for patients. In our review of previous research, we did not find research on multi-professional ethical competence or models that exist for multi-professional ethical practices, which motivates further research on ethical competence from a caring science perspective and the development of a model to strengthen a multi-professional ethical healthcare practice.

The idea of creating a proposed model in this research has its starting point in the thought that research knowledge is an important criterion for professionalism and the creation of theories and models to guarantee ethical professional practices.^12^ Models are broad statements that help the use of scientific knowledge in practices and professions to organize nursing care and offer higher quality care.^13,14^ Models should be used in clinical practice as a guide for the achievement of care goals and evaluation of outcomes. The implementation of such models contributes promotes individual and professional values, increases the motivation of work and quality of care.^12^ According to Nyholm et al.,^15^ the basis for sustainability in care practices depends on a clear expression of ethical values and the implementation of models for ethically accessible care in healthcare.

## Aim

This research aims to create a proposed model for multi-professional ethical competence grounded in clarified meanings and dimensions of ethical competence studied from a multi-professional healthcare perspective. The research questions are, what is ethical competence from a multi-professional healthcare perspective and what strengthens a multi-professional ethical healthcare practice?

## Research design

The present study is part of an ongoing collaborative research project between a university and university hospital in Norway with the aim of interweaving theoretical research and knowledge with current healthcare practice. A qualitative approach and hermeneutic application research methodology described by Koskinen and Nyström^16^ were chosen as methodology. Hermeneutic application research is a participatory-oriented methodology that has been developed to unite clinical practice and research theory. The basic idea is that participants with clinical experience and participants with theoretical backgrounds meet with the same premises and equivalents. The hermeneutic starting point was chosen to create room for reflective dialogues to attain new understanding.

### Recruitment of participants and data collection

Following the description of hermeneutical application research, the participants in this study consisted of three scientific researchers from the university and 13 multi-professional co-researchers from the university hospital. Recruiting-up of multi-professional co-researchers was done through an invitation to the entire healthcare personnel. The recruitment resulted in the formation of two equal dialogue groups with six participants from clinical practice and two scientific researchers in each. A total of four doctors, one psychologist, seven nurses and one care manager participated. The participants from clinical practice had backgrounds from various disciplines: psychiatry, surgery, emergency care, and internal medicine. In terms of age, three of the participants were between 20 and 30 years, two between 30 and 40 years, five between 40 and 50 years and two between 50 and 60 years. The variation in work experience was wide; three participants had worked from 1 to 5 years, three participants between 10 and 15 years, three participants between 15 and 20 years, two participants between 20 and 30 years and one participant between 30 and 35 years. Both men and women participated. A total of three scientific researchers with PhD degrees in health sciences and theoretical knowledge in caring science participated in and led the group dialogues. The scientific researchers had also a wide experience as caregivers, teachers, researchers or administrative leaders.

The groups each met four times for 2 h per time. Each time, the dialogue was started up with a short (10 min) introductory ethical theme grounded in Eriksson’s theory of caritative caring^17^ to initiate reflection, inspire dialogue and keep the focus of the dialogue on ethical issues. The ethical themes were human dignity, the caring encounter, ethical leadership and ethical competence. Participants were then given opportunities for personal reflection and open discussions about themselves as professionals and their care practices. The researchers acted as participants, but their task was also to create a relaxed and safe atmosphere and making sure that the conversation focused on the research object. During the dialogues, the participants reflected together on theories about ethics, how ethical values are expressed in clinical practice and finally together sought new understanding and possibly consensus about which ethical values are important for creating ethical practice from a multi-professional perspective, as well as what strengthens ethics and what challenges exist today for ethical practice. This allowed the scientific researchers an opportunity to reflect on ethical competence and ethical practice together with the co-researchers. The dialogues were recorded and transcribed into 117 pages of text (Times New Roman, 12 p, single-space).

### Data analysis

The transcribed text from the reflective dialogues was analysed through a thematic analysis according to Braun and Clarke.^18^ The thematization were conducted in five different steps. In the first step, the researchers familiarized themselves with the data. They read the interviews and listened to the audio files several times to obtain a more general impression of the material. In the next step, we read the data material again to verify meaning-bearing themes. As a third step, the themes were interpreted according to how they related to each other and how similarities and variations emerged in the material. The analysis in this phase resulted in six themes. Subsequently, the themes were arranged and clarified so that they highlighted the core after which the themes were reviewed concerning the whole. In the fourth step, the themes were defined and named based on their essence and content, and in the last step, the themes were compiled in a logical order to form the main pattern. The results are presented based on the themes and quotations.

## Ethical considerations

This study has been conducted according to the Declaration of Helsinki^19^ as regards ethical principles for research. The research project consulted the Norwegian Centre for Research Data (NSD)^20^ to ask for ethical permission, data management, and data protection. An ethical request was not needed as the participants were healthcare personnel. Consent for the study was also obtained from the participating healthcare organization’s upper management. The cover letter containing information about the study explained the purpose of the group dialogues and that participation in the study was voluntary and anonymity guaranteed. All participants signed informed consent. The data material has been stored according to the legislation regarding personal data and general data protection regulations. Ethical issues were considered throughout the process according to established ethical principles.

## Limitations

A limitation in this study may be that multi-professional competence is sought without covering all healthcare professionals. This is crucial in terms of model development and thus the proposed model in this research needs to be further tested in a broader multi-professional context. Hermeneutic application research belongs to so-called participatory action research designs and has also similarities with focus group interviews as a method. There are no unambiguous guidelines for the number of groups and when one can consider having achieved so-called saturation.^16,21^ The number of participants in this study completes the recommendation of a maximum of eight participants in each focus group interview,^22^ and it is a strength that the participants come from such varied professions and with such varied backgrounds. Implementation of ethical themes is in line with hermeneutic application research,^16^ but another limit for the study may be that the introductory themes have steered the dialogues in the desired direction. The transcribed interviews showed that the dialogues were initially about introductory themes but quickly turned into reflections concerning healthcare practice.

## Results

**Figure 1.**
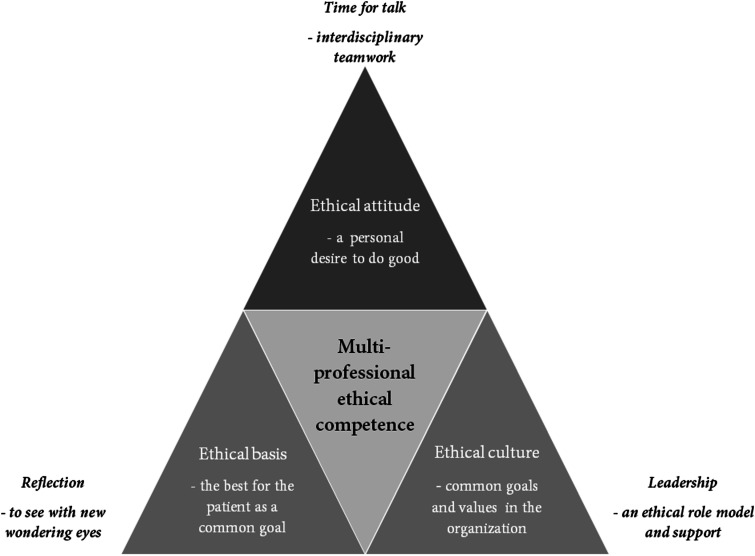
Proposed model for multi-professional ethical competence.

The proposed model (Figure 1) as an ideal model for multi-professional ethical competence encompasses a three-dimensional ethical value base that is underpinned by three themes; Ethical attitude – a personal desire to do good, Ethical basis – the best for the patient as a common goal and Ethical culture – common goals and values in the organization. What strengthens a multi-professional ethical competence is underpinned by three themes; Reflection – to see with new wondering eyes, Time for talk – interdisciplinary teamwork and Leadership – an ethical role model and support.

### Ethical attitude – a personal desire to do good

The starting point for all care is a will to do what is good, alleviate suffering and treat people with dignity and respect. Care is thus basically good, performed with the best of intentions, the best possible care for the patient, without imposing anything to which the patient does not agree. Therefore, it is important to consider our values, goals, rules, and our responsibility concerning ethical considerations. Ethics as action is something inherent in our personality, the way we behave, or as one participant said: *Healthcare personnel have and know the responsibility for ethics, maybe not on paper, but in your heart.*

The results highlight that the basis for an ethical attitude is sensitivity and discretion, empathy and compassion, and the ability for lifelong learning/practice. Sensitivity and discretion emerge especially in situations where healthcare professionals feel inadequate or it is difficult to reach the patient, or when judgment is required to understand what the patient needs. Doing what is best, then, requires being able to feel what it is like to be the other human being who is trying to convey something, and to ask oneself what is best there and then, what information should be given and how much and just do one’s best to provide care and convey consideration. Being sensitive means being able to see the person, to be present, listen quietly and try to understand the person completely and intuitively and in this way to adjust to being able to obtain a sense of how the other is feeling. In the words of a participant, *it is enough to be able to listen in and say to the patient, heavens what you are feeling pain and struggling*. Sensitivity and discretion, to be quiet, and facing how human beings are doing provides calmness in the meeting with the patient.

Empathy is about caring for other people and being able to empathize with how they feel, which requires not allowing oneself to be crushed and feeling defeated so that one cannot act or shed tears with the patient. Compassion is described as being present, touched by and open to the other person’s vulnerability and suffering and to show that one has understood. Compassion for the other person’s suffering is thus not the same as being readily affected by all patients because it can be tiring to be emotional. This is described as follows in the dialogues, *at some point in the conversation I realized, or it dawned on me, how much she is struggling… it was precisely that pain I suddenly felt myself, I almost felt that I started to cry, and it did something to both of us, and I think she felt that she could finally start talking about what was really the problem and we got closer to each other in a new way.*

Ethics is seen as a lifelong practice, not something one finishes, but a skill to treat and do the best for patients. It is not just knowledge, it is a desire to develop, wisdom to be more concerned with the patient than with oneself and an insight that it does something very good to oneself to meet the patient ethically. Ethics is a kind of ability that can further develop the humaneness in one’s professional competence.

### Ethical basis – the best for the patient as a common goal

The results highlight that the basis for all care and caring lies in a shared focus and main goal, namely what is best for the patient and what can be done by care personnel for the benefit of the individual patient. This is the basic task and motive for all work and why healthcare personnel have chosen to work in healthcare. Through the dialogues, two essential basic ideas emerged in doing the best for the patient: quality of life and seeing the patient as a unique human being. When it comes to quality of life, there is a determination that quality should be paramount all the way; quality for the individual patient should never be sacrificed because it is the basis for all care and caring. To always have the best for the patient and quality of life as a guiding light instead of just performing individual procedures and to remember that what is considered quality of life for oneself may not be so for the individual patient. It is not always straightforward, as one participant related, *I have to ask, what would the patient have wanted himself if he had a choice, what would we have wanted ourselves, what do we want for family members? Everything is only in our best judgment and preferably it should be from those closest to the patient*. Regarding the quality of life, it is important to discuss together and ask questions such as the following: should life end in this way when one puts on a respirator from time to time? Have the patients been allowed to say goodbye, have they talked with relatives and expressed what they want, as opposed to being put under anaesthesia and then it may not work? Is the patient prepared to die on the operating table? For what is the patient saved? What is important and what does the patient want to master in life? Quality of life emerges especially when it comes to dignity at the end of life. Thinking about the end of life has become unfamiliar to us, in one way people do not die anymore, and therefore it is important to safeguard a dignified and peaceful death instead of dutifully saving lives and following procedures, routines and rules.

To see patients as unique human beings means to respond to what is best for them, their desire to be treated as whole persons, and having a genuine interest for them as human beings. It includes having a holistic approach to patients, and of talking with them. At heart, it is about one’s view of human beings instead of seeing patients as a theory or that things should happen in a certain way. One of the participants described the importance of asking the questions what it is that makes the patients feel this way now, what is behind it, how their lives are, and what attitudes and what is important to them, instead of thinking that it is a difficult patient and situation. An ethical basis that says I want to see you as something more, I want to take part in your life. Seeing the patient can also mean a choice that involves acting against given routines. One of the participants described a situation in psychiatry where they were forced to bind a patient. In that situation, everyone in the care staff had received clear regulations to only focus on the procedure. In the described situation, the patient sought eye contact with his gaze and screamed, *see me dammit*. The decision to choose to attend and see the patient in such a situation requires breaking routines. This depends on how the individual is treated in the situation because everyone is different with different life baggage. Such a situation requires an ability to read the person using the tools one has so that the patient feels seen and has a sense of safety. It is not always the extra minutes that count, but their content. Ethics exist at the moment when one meets the patient on the same wavelength and is present.

Much is required at all levels; it is time-consuming and responsible to work in the patient’s best interests. The easiest is often to just press on with treatment because it is demanding and challenging to think about one’s values and point of view and to stop and think if this is the most beneficial for the patient. In the busy workday, the very focus is on following the procedures, not to exist for another human being.

### Ethical culture – common goals and values in the organization

Ethics as culture is how care is conducted, how caring is viewed, and the values of the organization. An ethical culture involves a shared way of thinking and attitude as an interdisciplinary team around the patient, thinking together for the patient. The main idea in an ethical culture is that everyone in the team has the same goal and does their part to ensure that the patient receives the best possible care. One participant gave an account of how important it is to think that when one enters an organization, one is bound and swallowed by that culture, so that one walks and trudges in it.

Attending to the patient and collegial dignity emerge as supporting themes when it comes to an ethical culture. In an ethical organizational culture, everyone must be concerned with taking care of and attending to the patient. The participants are concerned about unethical actions and quarrels in patient care. Today one often hears that this is not our patients and wonders where the patients should be and who owns them. This categorization does not belong to an ethical organizational culture because they are still patients regardless of illness and diagnosis. Collegial dignity means to appreciate each other and have respect for each other’s work, and thinking along the same lines, being on the same wavelength for everyone is in the same situation, nurses, doctors or psychologists, with similar issues daily and the same patients. To listen to and understand each other in the team is crucial because prejudices are reduced when hearing about other professionals’ experiences. This requires time and space and colleagues who are willing to listen, and that everyone speaks to each other in a dignified manner, treats and supports one another in the best possible way and dares to speak out in an openness where there is room for people to be themselves and they are allowed to feel vulnerable and such vulnerability can be seen as strength. It also requires frictionlessness, and that everyone is taken care of.

### Time for talk – interdisciplinary teamwork

Time and ethics go together, and it is therefore important to earmark time to discuss ethics, to sit down and go through what has been done well and what has not worked. Conversations within spaces where everyone is seen by the others and all share stories allow for the development of each participant in a profound way. Sometimes ethical talk is about discussing informal matters, at other times reflecting on a topic or ethical dilemmas in a specific patient situation. What is most important is that ethical discussions take place within the interdisciplinary team as teamwork around the patient, not in individual work, even if the individuals do exactly what they are supposed to do in the situation. Talking in interdisciplinary teams about situations gives more awareness, everyone is more involved in the decisions that are made, and the result is less automated actions.

Equally important is time to talk with patients and their relatives because they may have another motive. In these meetings, it is important to sit down, not be stressed in the situation because today health professionals are often under cross-pressure and at the centre of many views and then there is a risk that the patient’s voice suddenly disappears, because there are so many opinions that must be taken into account. Talking with patients and relatives is described as something that benefits oneself. A participant described the feeling like this, *you go home with a good feeling if, for example, when you can give the little extra and you notice that the other one is very happy about it. It is very nice; it gives an extra kick in the work*.

### Reflection – to see with new wondering eyes

Reflection occurs both in a multi-professional team and through self-reflection. Reflection means having the ability to self-reflect to learn about one’s own reactions because it does something to oneself, for better or worse, in work dealing with the ultimate consequences of life all the time. Self-reflection is an active choice and decision to think through and be honest with self. Self-reflection is to be conscious and constructively a little critical of oneself, is to be able to say that one is not happy with a certain situation or will not do it in the same way again and think for oneself how one should act next time. Reflection is also self-insight into one’s competence and respect for that one cannot or must know everything. Ethics is not to always have one answer to what is right and wrong, but reflection involves thinking about what one does, why one does it, and whether one is where one should be. Reflection is thinking about what was done right, if it could have been done differently, and learn from the experiences. As one participant put it: *That you sit back and think, oh horrible, what happened there. What was good and what was wrong because otherwise you drive straight ahead and do not think about what you are doing.*

Reflecting with colleagues is very important for standing together as a team in terms of collegial support, so that no one is left alone in a difficult situation. Therefore, it is important to prioritize natural breaks to have time for reflection to process and go through thoughts and experiences. Reflection in a multi-professional team entails going back to the situation and looking at how one acted at the time and gaining an opinion on how others see the situation and why it turned out the way it did. One participant thought that, *interdisciplinary reflection is very rewarding… and it is good that others come in with new wondering eyes and think something different. Reflecting on the ethical values in a work community is the basis for providing better care.*

### Leadership – an ethical role model and support

Ethics begins with the leader who thinks of the patients even though they are far from the organization’s leadership. The leader’s responsibility is to get staff involved in ethical thinking from a broad perspective and open up an understanding of ethics in the larger hospital context. Leaders must be clear in their ethical thinking and determined about how ethics is implemented in terms of treating everyone with the same dignity and respect, and also have the responsibility to back up the individual caregiver in difficult ethical situations. In administrative leadership, the focus can easily be only on operations. Therefore, it is valuable if a leader is close to practice to gain a better understanding of how care should work in the larger context and with the patient at the centre. One participant said, *it is not difficult to be ethical when standing by a patient, because you see the patient and know the need of the patient. As leaders, the same patient can easily become just a number*.

## Discussion

According to Milliken et al.,^4^ future research by using innovative methodologies is needed to understand the ethical nature of everyday practice. New understanding will help inform the development of tools to facilitate ethical awareness and sensitivity in all realms of nursing practice. The results in this hermeneutic application research highlight new understanding by a proposed model for multi-professional ethical competence in healthcare practice. Ethical competence is grounded in a three-dimensional ethical value base. Ethical competence is a personal ethical attitude and a personal desire to do good. Competence includes strength of character, ethical awareness, moral judgment and willingness to do good.^6^ Competence is a personal attitude, ability, action and lifelong exercise that includes healthcare personnel’s vulnerability, empathy, compassion and sensitivity that makes it possible to see, be present, touched, open and listen to the other person’s vulnerability and alleviate suffering. This result shows that ethical competence has a strong link to the core of caring ethics and a deeper personal value base, a caring ethos. Following Eriksson’s thinking,^17^ caring ethos is the characteristic of basic caring values that becomes visible in the caregiver’s attitude and action.

Ethical competence involves the whole culture and is seen as a shared way of thinking about values, goals, rules, given routines, and responsibility as a multi-professional team and organization. The desire and the will to do good and to do the best for the unique and vulnerable patient as a human being and the patient’s quality of life emerge as the basic ideas and core of ethical competence and culture. It is the healthcare personnel’s responsibility to always have the patient’s best and quality of life, instead of individual and organizational procedures, as a guiding light. Ethics involves thinking together and for the patient. Collegial dignity emerges in relation to one another as colleagues and includes respect for each other’s work and to think along the same lines. This is in line with Erikssons^17^ thought that ethos also creates the core of caring culture, an internal value hierarchy that requires values that are aimed at what is best for the patient. Healthcare personnel together with leaders as role models create the culture and important foundations and values that guide the entire care organization. The created model can contribute to a personal and also more organizational and cultural value base and so can constitute the ethos of the entire care organization. The results of this study highlight the need for more cooperation from a multi-professional perspective to understand each other’s professions regarding the complexity ethical competence and ethical culture entail in healthcare practice.

Leadership, reflection and time for talk emerge as methods for promoting ethical competence in practice. Leadership in a broad hospital context with the patient at the centre means to not just have the focus on operations, but to support ethical thinking in a practice that today often is affected by the cross-pressure that professionals experience. Hansen and Jørgensen^23^ highlights leadership that can promote ‘health humanization’ in terms of ‘authentic leadership’, ‘mindful leadership’ and ‘ethical leadership’, and argue that wonder-inspired leadership may enable to find the delicate balance between system-, task-, person- and phenomenon-led caring. The present study shows that different professions have a willingness and openness to talk and wonder about ethical issues in larger teams and that it is only by openness for talks and wondering reflections that different professions can recognize ethical challenges in daily activities from different professional perspectives, support and strengthen each other’s ethical competence and work towards common goals and create a shared ethical value base and good care for the patient. According to Nyholm et al.,^15^ it is through continuous discussions that the ‘rediscovering’ of ethical values in the culture is given new life. An ethical culture can be strengthened by meaningful dialogues, habits and routines in a multi-professional organization. To continuously reflect on and evaluate existing ethical values and routines can in turn increase work motivation and promote quality in care in healthcare practice.^12^

## Conclusion

The present article has presented the meaning and dimensions for multi-professional ethical competence in healthcare, and a proposed model has been created based on the results. The proposed model is a first step towards developing a theoretical model of multi-professional ethical competence and what strengthens a multi-professional ethical healthcare practice. The next step in the development of the model is to test and evaluate it multi-professionally in clinical practice. The goal of the model development is that it can be used as a tool, independent of context, to identify and strengthen the central ethical values necessary for a multi-professional ethical practice. A condition for how well the model in the future will be developed depends on how well the whole organization, managers and healthcare professionals continuously highlight and discuss the model and ethical values, and thereby maintain and strengthen their ethical competency.
